# Comparison of Neurodynamic Test Response Between Patients with Frequent Episodic Tension-Type Headache and Migraine: An Exploratory Comparative Study

**DOI:** 10.3390/jcm15208052

**Published:** 2026-10-16

**Authors:** Leandro H. Caamaño-Barrios, Ana Ostolaza-Guitian, Rubén Cuevas-Rodríguez, Juan Antonio Valera-Calero, Fernando Galán-del-Río, Sandra Sánchez-Jorge, Ricardo Ortega-Santiago

**Affiliations:** 1 Escuela Universitaria Gimbernat Cantabria, Adscrita la Universidad de Cantabria, 39300 Torrelavega, Spain; leandro.caamano@eug.es (L.H.C.-B.); ana.ostolaza@eug.es (A.O.-G.); ruben.cuevas@eug.es (R.C.-R.); 2 Department of Physiotherapy, Faculty of Nursery, Physiotherapy and Podiatry, Universidad Complutense de Madrid, 28040 Madrid, Spain; 3 Department of Physical Therapy, Occupational Therapy, Rehabilitation and Physical Medicine, Universidad Rey Juan Carlos, 28922 Alcorcón, Spain; fernando.galandel@urjc.es (F.G.-d.-R.); ricardo.ortega@urjc.es (R.O.-S.); 4 Grupo de Investigación de Alto Rendimiento en Evaluación Multidimensional y Tratamiento del Dolor Crónico, Universidad Rey Juan Carlos, 28922 Alcorcón, Spain; 5 Faculty of Health Sciences, Francisco de Vitoria University, 28040 Madrid, Spain

**Keywords:** migraine, neurodynamic, nerve tissues, sensitization, tension-type headache

## Abstract

**Background/Objectives:** Although migraine and frequent episodic tension-type headache (FETTH) may share sensory features, direct comparisons of responses to neurodynamic loading are scarce. Establishing whether responses differ may help distinguish shared from phenotype-specific features and guide future diagnostic research. The aim of this exploratory secondary analysis was to compare range-of-motion and pain responses during the Long Seated Slump Test (LSST), Seated Slump Test (SST), and Straight Leg Raise (SLR) in participants with migraine and FETTH and to explore their potential discriminatory performance. **Methods:** This cross-sectional observational secondary analysis included participants with FETTH (*n* = 31) and migraine (*n* = 33) classified by a neurologist according to ICHD-3 criteria. LSST outcomes were sacral flexion, cervical flexion, and pain at maximal cervical flexion; SST outcomes were active knee-extension angle and pain at maximal active knee extension in dominant and non-dominant limbs; and SLR outcomes were hip-flexion angle and pain intensity at a low-pain point (O_1_) and high-pain point (O_2_), assessed bilaterally with the ankle at 30° plantar flexion and neutral (0°). **Results:** In the unadjusted exploratory analyses, FETTH showed greater LSST sacral flexion than migraine (adjusted mean difference = 13.56°, 95% CI 8.58 to 18.54), whereas migraine showed greater pain at maximal LSST cervical flexion (adjusted mean difference = −1.45 NPRS points, 95% CI −2.42 to −0.48). An unadjusted between-group difference was also observed for the dominant-limb SLR angle at the high-pain point with the ankle neutral (adjusted mean difference = −10.17°, 95% CI −18.95 to −1.39). After Holm adjustment across the 23 neurodynamic ANCOVA comparisons, only LSST sacral flexion remained statistically significant. LSST sacral flexion also yielded the highest apparent AUC (0.881), with 78.8% sensitivity and 90.3% specificity at the same-sample exploratory cut-off. **Conclusions:** Neurodynamic responses differed selectively, with the clearest exploratory signal involving LSST sacral flexion. The same-sample cut-off is not a validated diagnostic threshold, and the sex imbalance between groups limits attribution to headache phenotype. Independent validation and assessment of incremental value beyond ICHD-3 classification are required before clinical use.

## 1. Introduction

Migraine and tension-type headache (TTH) are the two most prevalent primary headache disorders [1]. Although they are differentiated according to their clinical presentation, both encompass heterogeneous and sometimes overlapping phenotypes. Migraine is characterized by recurrent attacks of moderate-to-severe headache, commonly unilateral and pulsating, aggravated by routine physical activity, and accompanied by nausea and/or sensitivity to light and sound [2]. Some individuals also experience transient and fully reversible neurological symptoms known as migraine aura. In contrast, TTH typically presents as bilateral, pressing or tightening pain of mild-to-moderate intensity that is not aggravated by routine physical activity and is not accompanied by the prominent migrainous features described above [3]. Nevertheless, symptom overlap and coexistence between both disorders can complicate their differentiation, particularly when attacks are mild or do not display their complete clinical phenotype [4].

Both disorders represent major public health concerns. A comprehensive review of 357 population-based studies estimated the global prevalence of active TTH at 26.0% and that of migraine at 14.0%, although prevalence estimates varied considerably according to geographical region, study methodology, and reference period [5]. More recent Global Burden of Disease 2023 estimates indicate that approximately 2.9 billion people worldwide are affected by headache disorders. TTH remains more prevalent than migraine, with an age-standardized prevalence of approximately 24.9%, whereas migraine affects approximately 14.1% of the global population [6]. Despite its lower prevalence, migraine accounts for most headache-attributable disability, and the burden associated with headache disorders is more than twice as high in women as in men [6]. These findings highlight the need not only to characterize both disorders accurately but also to identify clinical features that may contribute to their differentiation and improve the selection of disorder-specific management strategies.

Migraine and TTH are currently regarded as distinct but partially overlapping disorders involving altered nociceptive processing [7]. Activation and sensitization of the trigeminovascular system are central to migraine pathophysiology, whereas TTH is thought to involve a variable contribution of peripheral myofascial nociception and central sensitization, with the relative contribution of these mechanisms differing according to headache frequency and chronicity [8,9]. Accordingly, both disorders may be associated with local and widespread sensory alterations. Recent systematic-review evidence indicates that individuals with migraine or TTH can exhibit cervical musculoskeletal impairments and increased pain sensitivity [10,11], although the magnitude and consistency of these findings vary substantially across clinical subgroups and assessment procedures [12]. The presence of extra-cephalic sensory responses also suggests that physical examination should not necessarily be restricted to symptomatic cranial or cervical structures [13]. These differences provide a biologically plausible reason to compare the two phenotypes under a standardized mechanical challenge, but they do not imply that migraine or FETTH should produce a disease-specific response in a distant limb. Neurodynamic procedures combine spinal, limb, neural, muscular, fascial, and behavioral components. Therefore, any between-group difference would be interpreted as a phenotype-associated response to the movement sequence rather than as a direct measure of neural mechanosensitivity or pathology [14].

Neurodynamic tests provide one possible approach for examining responses to controlled mechanical loading across different components of the nervous and musculoskeletal systems [15]. These tests involve sequential joint and spinal movements intended to modify the mechanical environment of neural structures. Their interpretation may incorporate range of motion, the onset and intensity of provoked symptoms, symptom distribution, and changes produced by structural differentiation. However, because neurodynamic tests also load non-neural tissues and can provoke responses in asymptomatic individuals, their findings should be interpreted as indicators of mechanosensitivity within the tested movement sequence rather than as direct evidence of neural pathology [16]. The Long Seated Slump Test (LSST) was included because it combines lumbopelvic/trunk flexion with a fixed lower-limb position and provides a measurable proximal movement outcome (sacral flexion) before cervical flexion is added. This outcome was intended to describe the participant’s lumbopelvic movement response within the whole test sequence, not to serve as a surrogate for neural tissue length or pathology. Its inclusion was also supported by prior headache research using the Seated Slump Test (LSS)/LSST and related tests [16,17]. The SST [18] and Straight Leg Raise (SLR) [19] were included as complementary procedures because they apply different combinations of spinal and lower-limb mechanical loading, allowing movement and symptom responses to be examined across distinct neurodynamic sequences. Together, the LSST, SST, and SLR were selected to provide a complementary characterization of responses to mechanical loading, supported by their previous use in neurodynamic and headache research [14,15,16], rather than because they represent established diagnostic tests for primary headache.

Evidence regarding neurodynamic responses in headache disorders remains limited. In children, greater sensory responses during the LSST have been observed in participants with migraine or cervicogenic headache compared with headache-free controls [20]. In adults with side-dominant head or neck pain, altered responses during an occipital nerve neurodynamic test have also been reported [17]. More directly relevant to TTH, women with frequent episodic TTH (FETTH) demonstrated reduced range of motion during the SLR, SST, and LSST and greater symptom responses during some of these procedures compared with headache-free women [21]. Nevertheless, the available studies have mainly compared individual headache populations with asymptomatic controls, and neurodynamic findings remain insufficiently investigated in migraine. However, no previous study has directly compared neurodynamic responses between adults with migraine and FETTH or examined whether findings obtained from these tests can discriminate between the two conditions, even though such a comparison may help determine whether altered responses to mechanical loading represent a shared feature of primary headache disorders or whether specific neurodynamic findings differ according to headache phenotype. Although clinical classifications are diagnosed according to established ICHD-3 criteria, quantifying the discriminative performance of neurodynamic findings remains informative in a known-group design and complements information on between-group differences and may serve as adjunctive markers for clinical characterization, rather than as alternative diagnostic criteria or stand-alone diagnostic tests.

Therefore, the main aim of this study was to compare range-of-motion and pain responses during the LSST, SST, and SLR tests between participants with migraine and FETTH. A secondary, exploratory aim was to quantify, using ROC analysis, the discriminative separation provided by these neurodynamic findings between the clinically defined migraine and FETTH groups, as a complement to the between-group comparisons.

## 2. Materials and Methods

### 2.1. Study Design

This is a cross-sectional observational study designed to compare neurodynamic test responses in a sample of patients with FETTH and a sample suffering from migraine recruited from one university-based center. Participants were allocated to the corresponding headache group after a structured clinical assessment performed by an experienced neurologist following the ICHD-3 diagnostic criteria and predefined eligibility criteria. Methodological reporting followed the Strengthening the Reporting of Observational Studies in Epidemiology (STROBE) statement [22] and the Enhancing the QUAlity and Transparency Of health Research (EQUATOR) recommendations [23].

Because the present work is a secondary analysis, the LSST, SST, and SLR outcomes were drawn from the neurodynamic measures available in the parent dataset. No single test or outcome was designated as a primary diagnostic marker before this analysis.

### 2.2. Participants

Potential volunteers with a clinical diagnosis of migraine or episodic TTH were consecutively recruited from a university-based center in Cantabria, Spain. Diagnostic classification was established by an experienced neurologist, who served as the clinical reference, and migraine was identified according to the third edition of the International Classification of Headache Disorders [3]. All participants provided written informed consent before inclusion in the study. No formal a priori sample-size calculation was performed for this secondary analysis. The sample comprised all eligible participants with complete data for the variables required for the present analyses.

#### 2.2.1. Eligibility Criteria for Patients with Migraine

Clinical information related to migraine was systematically collected, including headache location and characteristics, duration of the disorder, frequency and intensity of attacks, family history, and medication use. Participants were excluded in the presence of chronic migraine; migraine with aura; another primary or secondary headache disorder, including medication-overuse headache; a history of head or cervical trauma, such as whiplash injury; pregnancy; cervical disc herniation; clinically relevant systemic or musculoskeletal comorbidities, such as rheumatoid arthritis; other persistent pain disorders, including fibromyalgia or cancer-related pain; or any medical procedure performed during the previous six months or any undergoing pharmacological or non-pharmacological preventive therapy capable of modifying headache symptoms, pain sensitivity, or musculoskeletal/neurodynamic responses (such as local anesthetic or corticosteroid injections, botulinum toxin injections, or surgery involving the head, cervical spine, or other regions relevant to the neurodynamic assessment). Assessments were performed during the interictal period, outside an active migraine episode. Female participants were also required not to be menstruating on the day of assessment.

#### 2.2.2. Eligibility Criteria for Patients with FETTH

Participants allocated to the FETTH group were required to have experienced at least 10 headache episodes occurring on 1–14 days per month on average for >3 months (≥12 and <180 days per year), with individual episodes lasting from 30 min to 7 days and a pressing or tightening quality [3]. Complementary to these ICHD-3 criteria, participants were required to present mild-to-moderate intensity, corresponding to 3–6 points on a 0–10 numerical pain rating scale [24]. Headache was not permitted to worsen with routine physical activity and had to occur without nausea or vomiting, although either photophobia or phonophobia could be present. 

Participants were excluded if they presented with chronic TTH, another primary or secondary headache disorder, fibromyalgia, cervical disc herniation, or cervical osteoarthritis based on clinical history, previous head or neck trauma, systemic degenerative disease, pregnancy, or if they had undergone anesthetic block procedures or received physical therapy during the preceding six months.

### 2.3. Neurodynamic Tests

A single assessor with more than 10 years of clinical experience and blinded to the participants’ classification performed all tests. The LSST, SST, and SLR neurodynamic tests were standardized and used to examine participants’ responses to mechanical loading, in that order. Before data collection, participants received standardized instructions and completed a familiarization procedure to ensure that they understood the test sequence and the pain-intensity thresholds used during the assessment. Each measurement was performed three times, with a 30 s rest period between repetitions to minimize potential carry-over effects from the preceding assessment (e.g., temporal summation of pain, fatigue, or transient changes in pain or range-of-motion responses), and the mean of the three trials was used for the statistical analysis. For bilateral tests, the order in which the dominant and non-dominant lower limbs were assessed was randomized.

#### 2.3.1. Long Seated Slump Test

The LSST was performed following the procedure described by von Piekartz et al., who reported high reliability for repeated measurements (with ICC values ranging from 0.89 to 0.99) [20]. Participants sat on the examination table with both lower limbs extended, the knees maintained in full extension, and the ankles dorsiflexed. A stabilization strap was positioned approximately 10 cm proximal to the superior border of the patella to maintain contact between the posterior aspect of the knees and the examination table.

From this position, participants were instructed to actively flex the thoracic and lumbar spine as far as comfortably possible. Sacral flexion was measured in degrees using a handheld inclinometer (values above the horizontal reference in the direction of anterior sacral inclination were recorded as positive, whereas values beyond the horizontal reference in the opposite, posterior direction were recorded as negative) while the participant maintained the maximal spinal-flexion position for 5 s. Participants were then asked to add maximal active cervical flexion while maintaining the position of the trunk and lower limbs. Cervical flexion was quantified using a cervical range-of-motion device, and the intensity of pain experienced at maximal cervical flexion was recorded using an 11-point Numeric Pain Rating Scale (NPRS), ranging from 0 (“no pain”) to 10 (“worst imaginable pain”) [25]. The mean sacral-flexion angle, cervical-flexion angle, and pain intensity obtained across the three repetitions were used in the analysis.

#### 2.3.2. Seated Slump Test

The SST was conducted using a standardized sequence of spinal and lower-limb movements [18]. Participants sat on the edge of the examination table with the knees together and the popliteal creases positioned at the edge of the table. They were initially instructed to place their hands behind their back and actively slump the thoracic and lumbar spine. The examiner monitored the cervicothoracic region to ensure that the required spinal position was maintained. Participants then performed craniocervical flexion by bringing the chin towards the sternum, after which the examiner stabilized the cervical position. The ankle of the limb being examined was subsequently moved into maximal dorsiflexion and maintained in this position while the participant actively extended the knee as far as possible.

The active knee-extension angle achieved at the end of the movement was measured in degrees. Pain intensity at maximal active knee extension was recorded using the 0–10 NPRS. The procedure was performed separately for the dominant and non-dominant lower limbs, and the order of limb assessment was randomized. Three trials were completed for each limb, and the mean active knee-extension angle and pain-intensity score were used in the analysis. Previous studies have reported high intra- and inter-examiner reliability for measurements obtained during the Slump Test (with correlation coefficients of 0.95 and 0.92, respectively) [26].

#### 2.3.3. Straight Leg Raise Test

The SLR test was used to assess responses to progressive mechanical loading of the lumbosacral nerve roots and lower-limb neural structures. The procedure was based on the protocol described by Boyd et al. [19]. Participants were positioned supine with both lower limbs relaxed and extended. A gravitational inclinometer was secured immediately distal to the tibial tuberosity of the limb being assessed to quantify the hip-flexion angle.

The examiner passively raised the lower limb into hip flexion while maintaining the knee in full extension. The test was performed under two ankle conditions: 30° of plantar flexion and a neutral ankle position (0° of plantar flexion). The ankle was maintained in each predefined position using a positioning splint. The neutral position was used to increase mechanical loading relative to the plantar-flexed condition, as ankle dorsiflexion increases the mechanical contribution of the neural structures during the SLR test [19].

For each ankle position, the hip-flexion angle and pain intensity were recorded at two predefined points. The first point, O_1_, corresponded to the low-pain observation point, defined as a pain intensity of 0–3 points on the NPRS. The second point, O_2_, corresponded to the high-pain observation point, defined as an intensity of 8–10 points on the NPRS. Participants were required to maintain the O_2_ position for approximately 5 s while the corresponding hip-flexion angle and pain-intensity score were recorded.

Both ankle conditions were assessed in the dominant and non-dominant lower limbs. Three trials were performed for each combination of limb, ankle position, and pain threshold, with a 30 s rest period between trials. The mean SLR angle and NPRS score at O_1_ and O_2_ were used in the statistical analysis. Handheld inclinometry has demonstrated appropriate reliability for quantifying hip-flexion range of motion during SLR neurodynamic testing (with ICCs ranging between 0.78 and 0.96) [27].

### 2.4. Clinical and Headache Characteristics

Demographic and headache-related characteristics were recorded, including age, sex, headache history, headache frequency, and mean pain intensity measured with a NPRS [24]. Individual 0–10 numerical pain-intensity ratings have demonstrated adequate reliability and validity for group-level pain research. In chronic pain populations, test-retest stability coefficients for individual 0–10 ratings have ranged from approximately 0.55 to 0.84, with a median of 0.72, and these measures have demonstrated responsiveness to expected treatment-related changes in pain intensity. Numerical rating scales also show good correspondence with other established unidimensional pain measures and are widely supported for assessment of pain intensity in adults [28].

Headache-related disability was assessed using the Headache Disability Index (HDI), which provides a total score ranging from 0 to 100, with higher scores indicating greater headache-related disability [29]. The original HDI demonstrated high internal consistency (Cronbach’s α ≈ 0.94), with short-term test-retest stability over approximately 1 week of r = 0.93–0.95 and longer-term stability over approximately 2 months of r = 0.76–0.83. Construct validity has been supported by the relationship between HDI scores and headache severity and by generally congruent patient and spouse ratings of headache-related disability [30].

### 2.5. Statistical Analysis

Statistical analyses were conducted using the Statistical Package for the Social Sciences (SPSS), v.31 for Mac OS (IBM Corp., Armonk, NY, USA). All analyses were performed using two-tailed tests. Continuous variables are reported as mean and standard deviation. Because no single neurodynamic outcome had been prespecified as primary in this secondary analysis, the between-group analyses were considered exploratory. Accordingly, interpretation focused primarily on effect estimates and their 95% confidence intervals rather than on dichotomous interpretation of individual unadjusted *p* values.

Because the FETTH and migraine groups differed significantly in age, headache duration, and HDI, between-group comparisons of neurodynamic outcomes were performed using analysis of covariance (ANCOVA), with headache group entered as the fixed factor and age, headache duration, and HDI included as continuous covariates. Given the unequal sex distribution between groups, the potential influence of sex on each neurodynamic outcome was additionally explored within the migraine group using independent-samples comparisons between male and female participants. Sex was subsequently included as an additional fixed factor only in ANCOVA models for outcomes showing a statistically significant sex-related difference within the migraine group (*p* < 0.05).

For each model, adjusted mean differences between FETTH and migraine, together with their 95% confidence intervals, F statistics, unadjusted *p* values, and partial eta squared (ηp^2^), were calculated. Adjusted differences were expressed as FETTH minus migraine. To assess the robustness of the findings to multiplicity, a sensitivity analysis was performed using the Holm procedure across all 23 neurodynamic ANCOVA comparisons. Holm-adjusted *p* values were therefore interpreted as the multiplicity-adjusted sensitivity analysis, while unadjusted *p* values were retained to describe the exploratory analyses.

Receiver operating characteristic (ROC) analyses were conducted as exploratory same-sample derivation analyses to describe the discriminative separation provided by individual neurodynamic findings between participants with migraine and FETTH. Migraine was defined as the positive condition and FETTH as the negative condition. For each outcome, the area under the ROC curve (AUC) and its 95% confidence interval were calculated. The threshold maximizing Youden’s index (sensitivity + specificity − 1) within the present sample was identified solely to describe sample-specific operating characteristics and was not considered a validated diagnostic cut-point. Sensitivity and specificity at each sample-derived threshold were reported with 95% exact binomial confidence intervals. Positive and negative likelihood ratios (LR+ and LR−) were also reported with 95% confidence intervals derived from the corresponding 2 × 2 classification tables. When no false-positive observations occurred, the LR+ point estimate was infinite, and the corresponding confidence interval was reported with an infinite upper bound.

For variables in which lower rather than higher values were associated with migraine, the direction of the ROC curve was reversed so that the reported AUC represented discrimination in the relevant direction. The direction of the corresponding sample-derived classification threshold was modified accordingly. Because threshold selection and performance estimation were conducted in the same sample and no internal resampling validation was performed, all ROC-derived operating characteristics represent apparent, exploratory estimates and should not be interpreted as validated diagnostic performance.

## 3. Results

Seventy-five consecutive volunteers willing to participate and reporting headache were screened for eligibility criteria. Eleven participants were excluded: chronic tension-type headache (*n* = 6) and previous whiplash (*n* = 5). No missing data were present for the variables included in the main analyses. Therefore, complete-case analyses were performed, including a total of 64 participants, divided into a FETTH group (*n* = 31) and a migraine group (*n* = 33, 100% without aura). Table 1 summarizes the demographic and clinical characteristics of both groups. Participants with migraine were slightly but significantly older than those with FETTH (*p* = 0.032), and sex distribution differed between groups, with all participants in the FETTH group being female compared with 69.7% in the migraine group (*p* < 0.001). Participants with migraine also reported a longer history of headache (*p* = 0.011) and greater headache-related disability, as reflected by higher Headache Disability Index scores (*p* = 0.009). In contrast, no statistically significant between-group differences were observed for the number of headache episodes per month (*p* = 0.066) or mean pain intensity per attack (*p* = 0.846).

Table 2 summarizes neurodynamic test responses in participants with FETTH and migraine, together with the adjusted between-group comparisons. All analyses were adjusted for age, headache duration, and HDI, and sex was additionally included in the model for outcomes showing significant sex-related differences within the migraine group. Because no outcome had been prespecified as primary, these comparisons were considered exploratory.

In the unadjusted exploratory analyses, three outcomes had *p* values below 0.05. Sacral flexion during the LSST was greater in participants with FETTH than in those with migraine (adjusted difference = 13.56°, 95% CI 8.58 to 18.54; F = 29.69; unadjusted *p* < 0.001; ηp^2^ = 0.331). Participants with migraine also reported greater pain intensity at maximal cervical flexion during the LSST (adjusted difference = −1.45 NPRS points, 95% CI −2.42 to −0.48; F = 8.94; unadjusted *p* = 0.004; ηp^2^ = 0.128). Cervical flexion itself showed greater uncertainty around the between-group estimate and did not meet the nominal *p* < 0.05 threshold (*p* = 0.094). After Holm adjustment across the 23 neurodynamic comparisons, only LSST sacral flexion remained statistically significant (Holm-adjusted *p* < 0.001), whereas pain at maximal cervical flexion did not (Holm-adjusted *p* = 0.088).

For the SST, neither active knee-extension range nor pain at maximal active knee extension showed a robust between-group difference. The unadjusted *p* values for pain were 0.058 and 0.061 for the dominant and non-dominant limbs, respectively, and all Holm-adjusted *p* values for SST outcomes were 1.000.

For the SLR, most between-group estimates were compatible with substantial overlap between groups across limbs, ankle positions, and pain observation points. The dominant-limb SLR angle at the high-pain point (O2) with the ankle neutral showed an unadjusted between-group difference (adjusted difference = −10.17°, 95% CI −18.95 to −1.39; F = 5.37; unadjusted *p* = 0.024; ηp^2^ = 0.082). However, this isolated finding did not remain statistically significant after Holm adjustment (adjusted *p* = 0.503). No other SLR comparison had an unadjusted *p* value below 0.05. Overall, the multiplicity-adjusted sensitivity analysis therefore identified LSST sacral flexion as the only between-group finding robust to correction for multiple comparisons.

Table 3 summarizes the exploratory same-sample ROC derivation analyses. Sacral flexion during the LSST showed the highest apparent discriminative performance, with an AUC of 0.881 (95% CI 0.797–0.965; *p* < 0.001). Because lower sacral-flexion values were associated with migraine, the direction of the ROC curve was inverted. The sample-derived threshold maximizing Youden’s index was ≤−1.00°. At this threshold, sensitivity was 78.8% (95% CI 61.1–91.0), specificity was 90.3% (95% CI 74.2–98.0), LR+ was 8.12 (95% CI 3.07–24.00), and LR− was 0.23 (95% CI 0.12–0.46). Because both the threshold and its operating characteristics were derived from the same sample, these estimates represent apparent performance and should not be interpreted as a validated diagnostic threshold.

Pain intensity at maximal cervical flexion during the LSST yielded an AUC of 0.712 (95% CI 0.587–0.838; *p* = 0.001). The sample-derived threshold of ≥7.85 NPRS points provided a sensitivity of 81.8% (95% CI 64.5–93.0) and specificity of 51.6% (95% CI 33.1–69.8), with an LR+ of 1.69 (95% CI 1.17–2.63) and LR− of 0.35 (95% CI 0.16–0.78). Pain at maximal active knee extension of the dominant limb during the SST yielded an AUC of 0.652 (95% CI 0.514–0.790; *p* = 0.031). At the sample-derived threshold of ≥6.25 NPRS points, sensitivity was 78.8% (95% CI 61.1–91.0), specificity was 54.8% (95% CI 36.0–72.7), LR+ was 1.74 (95% CI 1.17–2.78), and LR− was 0.39 (95% CI 0.19–0.80).

The remaining ROC analyses generally showed more limited apparent discrimination. For the SLR outcomes, AUC estimates were relatively close to 0.50, and their 95% confidence intervals included 0.50. Some sample-derived thresholds produced relatively high specificity and correspondingly large or infinite LR+ point estimates; however, these were accompanied by low sensitivity and wide confidence intervals. For example, the dominant-limb O2 angle at 0° plantar flexion yielded an AUC of 0.623 (95% CI 0.481–0.765; *p* = 0.090), whereas the corresponding non-dominant-limb measure yielded an AUC of 0.626 (95% CI 0.485–0.767; *p* = 0.079). These estimates should therefore be interpreted cautiously and as hypothesis-generating rather than as evidence of validated discriminatory thresholds.

In general, the ROC and adjusted between-group analyses showed the clearest convergence for sacral flexion and pain during maximal cervical flexion in the LSST, although the two analytical approaches did not yield identical patterns across all outcomes. The ANCOVA models accounted for age, headache duration, and HDI and, for selected outcomes, sex, whereas the ROC analyses evaluated unadjusted individual measures. Furthermore, the ROC thresholds and their operating characteristics were derived and evaluated in the same sample. Accordingly, the ROC findings should be considered exploratory derivation results rather than confirmation of a stable diagnostic pattern.

## 4. Discussion

The most important finding was that responses to neurodynamic loading differed selectively rather than globally between migraine and FETTH. The clearest between-group difference was observed in sacral flexion during the LSST, which was the only neurodynamic outcome that remained statistically significant after Holm adjustment. Although pain at maximal cervical flexion during the LSST and the dominant-limb SLR angle at O_2_ with the ankle neutral had unadjusted *p* values below 0.05, neither remained statistically significant after multiplicity adjustment. These findings should therefore be considered hypothesis-generating signals rather than independent evidence of phenotype-specific differences. Most SST and SLR outcomes showed substantial overlap between groups. The marked sex imbalance between groups should also be considered when interpreting the main finding: all participants with FETTH were women, whereas approximately 30% of the migraine group were men. Although sex was included in the ANCOVA model for sacral flexion because a sex-related difference was identified within the migraine group, statistical adjustment cannot fully separate the effects of sex and headache phenotype when one group contains no men. Accordingly, the observed LSST difference cannot be attributed exclusively to headache phenotype.

The difference in sacral flexion during the LSST may have several plausible, non-mutually exclusive explanations. The LSST is a composite movement task in which lumbopelvic and trunk flexion occur while the knees remain extended and the ankles dorsiflexed. Consequently, the final sacral position may reflect differences in lumbopelvic contribution to the movement, mobility, or stiffness of neural and non-neural tissues, motor strategy, or tolerance to the combined movement sequence. Neurodynamic procedures alter the mechanical environment of neural structures but simultaneously load muscular, fascial, and other musculoskeletal tissues [15,16]. The present protocol did not quantify the individual contribution of these structures or include structural differentiation; therefore, the observed sacral difference cannot be assigned to a specific biomechanical mechanism. It is more appropriately interpreted as a difference in the movement response elicited by the LSST rather than as direct evidence of neural pathology or peripheral nerve sensitization.

Sacral flexion during the LSST showed the highest apparent AUC in the exploratory ROC derivation analysis. This finding identifies a potentially relevant variable for future replication, but the sample-derived threshold and its operating characteristics were estimated in the same participants and from an individual, unadjusted measure. It should therefore not be regarded as a validated diagnostic threshold or as evidence that the test improves clinical classification beyond the established diagnostic assessment. The relatively large between-group effect for sacral flexion (ηp^2^ = 0.331) should likewise be interpreted within the present sample and requires independent replication. In addition, the sample-derived threshold of ≤−1° lies very close to the horizontal reference (0°) used for the inclinometer measurement. Its exact numerical value may therefore partly reflect the measurement convention and characteristics of the LSST procedure rather than a biologically meaningful boundary between headache phenotypes. The greater pain reported during maximal cervical flexion in participants with migraine should likewise be interpreted as a difference in symptom response to the test procedure rather than as evidence of a specific mechanism. The present design cannot determine whether this response reflects sensory processing, mechanical factors, behavioral responses, pain expectations, symptom tolerance, or other mechanisms. Moreover, this finding did not remain statistically significant after multiplicity adjustment and should therefore remain hypothesis-generating.

The isolated SLR O_2_ finding warrants particularly cautious interpretation. Although an unadjusted between-group difference was observed for the dominant limb with the ankle neutral, a comparable pattern was not consistently reproduced in the contralateral limb or across the other loading conditions, and the finding did not remain statistically significant after Holm adjustment. In addition, the corresponding ROC analysis showed limited apparent discrimination. Taken together, the lack of consistency across related SLR outcomes and the attenuation of the result after multiplicity adjustment make sampling variability a plausible explanation. Therefore, the present data do not provide evidence of a reproducible difference in SLR response between migraine and FETTH.

Direct evidence regarding neurodynamic responses in headache disorders remains limited. The closest comparator is the case-control study by Caamaño-Barrios et al. [21], which reported reduced movement responses during the SLR, SST, and Long Sitting Slump Test, together with greater pain responses during the latter two procedures in women with FETTH compared with headache-free women. These findings support the possibility that some responses to mechanical provocation differ between FETTH and asymptomatic participants. However, they do not establish the direction or magnitude of differences between FETTH and migraine. The samples are not independent, as the 31 participants with FETTH included in the present secondary analysis were also included in the previously published FETTH cohort [21]. The previous study addressed whether neurodynamic responses differed between women with FETTH and headache-free controls, whereas the present secondary analysis addresses a different research question by directly comparing participants with FETTH and migraine and by exploring the discriminative separation of neurodynamic measures between these two clinically defined headache groups. The previous report should therefore be considered methodological and descriptive context rather than an independent replication of the present findings.

In children aged 6–12 years, von Piekartz et al. [20] reported greater sensory-response intensity during the Long Sitting Slump Test in participants with migraine or cervicogenic headache than in controls. Leg responses were particularly frequent in the migraine group, whereas no migraine-control difference was found for sacral position, cervical flexion, or standardized knee flexion. The greater pain response during the LSST observed in the present migraine group is partially compatible with this pattern. Nevertheless, the comparison is limited by the different age range, comparator group, and operational definitions of the test outcomes.

Zito et al. [30] studied adults with cervicogenic headache, migraine with aura, and headache-free controls using a broader cervical musculoskeletal examination. Most measures were similar between the migraine and control groups, while the clearest abnormalities were observed in the cervicogenic headache group. This contrasts with the present LSST findings and suggests that results may depend on the headache phenotype, the specific test procedure, and the outcome selected rather than reflecting a uniform neurodynamic profile across headache disorders.

Szikszay et al. evaluated the occipitalis Long Sitting Slump Test and greater occipital nerve pressure sensitivity in adults with side-dominant head and neck pain, including participants with episodic or chronic migraine and cervicogenic headache [17]. They reported greater pain responses during the occipital test and lower pressure pain thresholds over the greater occipital nerve than in healthy controls. However, the mixed clinical sample and the use of an occipital rather than a lumbosacral neurodynamic procedure limit direct comparison with the present study. Their findings may support altered cranial or occipital mechanosensitivity in selected headache populations, but they do not establish the ability of these measures to distinguish migraine from FETTH.

Taken together, previous studies suggest that altered responses to mechanical or neurodynamic provocation may occur in some headache populations, but the findings are heterogeneous and appear to depend on the clinical phenotype and testing protocol. The exploratory ROC analyses in the present study should also be distinguished from clinical diagnostic utility. They quantify how well individual measures separated participants who had already been classified as migraine or FETTH according to ICHD-3 criteria within this particular sample. Statistical separation between known groups (even when accompanied by an apparently favorable AUC, sensitivity, or specificity) does not demonstrate that a measure can diagnose an undifferentiated patient in clinical practice. Establishing diagnostic utility would require prospective evaluation in an appropriate clinical population, independent validation of the derived thresholds, and evidence that the test provides clinically meaningful information beyond established diagnostic criteria. These conditions were not evaluated in the present study. Accordingly, the ROC results represent exploratory statistical discrimination rather than evidence of an additional diagnostic test.

### 4.1. Clinical Implications

From a clinical perspective, the present findings do not support the use of the LSST, SST, or SLR as stand-alone tests for differentiating migraine from FETTH. The clearest exploratory finding was the difference in sacral response during the LSST, suggesting that the two groups may respond differently to this combined lumbopelvic, spinal, and lower-limb movement challenge. However, its underlying mechanism remains uncertain; the sex imbalance limits attribution solely to headache phenotype, and the finding requires independent replication. The sample-derived ROC threshold should therefore not be used for individual diagnosis or treatment decisions. At present, neurodynamic testing may instead provide complementary descriptive information about movement and symptom responses during physical examination. Whether these responses add clinically useful information beyond ICHD-3 classification, predict prognosis, or inform treatment selection remains unknown and requires prospective investigation.

### 4.2. Limitations

Several limitations should be considered. First, this was a relatively small, single- Several limitations should be considered. First, this was a cross-sectional secondary analysis from a single university-based center, with a relatively small and young sample and no a priori sample-size calculation. The clinical spectrum may therefore be narrower than that encountered in routine practice. Differences between groups in age, sex distribution, headache history, and headache-related disability may also have influenced the neurodynamic responses. Although adjusted analyses were conducted and sex was explored in selected models, residual confounding cannot be excluded, particularly because the absence of men in the FETTH group prevents the independent effects of sex and headache phenotype from being fully disentangled. The generalizability of the findings to other migraine phenotypes, chronic headache populations, and men is consequently uncertain.

Second, the LSST, SST, and SLR are composite movement procedures that may be influenced by neural, muscular, fascial, spinal, and behavioral factors. The protocol did not establish that the responses reproduced the participants’ typical headache symptoms or that they changed with a formal structural-differentiation maneuver. Accordingly, the study cannot determine the specific tissue or mechanism responsible for the observed responses. The observed differences should therefore not be interpreted as evidence of isolated neural mechanosensitivity. Relevant factors such as medication use, psychological variables, and other aspects of migraine phenotype may also have contributed to the responses. In addition, although 30 s rest periods were incorporated between repetitions to minimize carry-over effects, repeated high-intensity symptom provocation at O_2_ during the SLR could potentially have influenced subsequent measurements through transient temporal summation or other carry-over effects; this possibility cannot be completely excluded.

Finally, numerous related neurodynamic outcomes were examined, increasing the probability of chance findings. To address this issue, a Holm-adjusted sensitivity analysis was performed across the 23 ANCOVA comparisons. The results derived from Holm adjustments reinforce the exploratory nature of the analyses and the importance of interpreting the findings primarily through the magnitude and precision of the effect estimates rather than isolated unadjusted *p* values. The ROC analyses remained exploratory and were based on unadjusted individual measures, while the cut-offs were selected within the same sample using Youden’s index. Because these ROC analyses were unadjusted, part of the apparent discrimination may reflect the baseline differences in age and sex distribution between groups rather than headache phenotype alone. This may produce optimistic estimates of discrimination, and the likelihood ratios, particularly infinite values resulting from zero false-positive observations, are unstable in a sample of this size. No internal resampling or external validation was performed. Accordingly, the proposed thresholds should not be applied clinically until they have been tested prospectively in larger, balanced, and independently recruited cohorts.

## 5. Conclusions

This exploratory secondary analysis identified LSST sacral flexion as the clearest between-group difference between participants with migraine and FETTH and the only ANCOVA finding that remained statistically significant after Holm adjustment for multiple comparisons. Other LSST, SST, and SLR findings were largely overlapping or did not remain statistically significant after multiplicity adjustment. LSST sacral flexion also showed the strongest exploratory discrimination, but this finding does not establish neural pathology or a clinically validated diagnostic threshold. Interpretation is further limited by the all-female FETTH group and the partial sex imbalance in migraine. Observed differences cannot be attributed solely to headache phenotype, and these findings do not demonstrate added diagnostic value beyond ICHD-3 classification. Independent, sex-balanced validation studies with prespecified outcomes and external assessment of incremental value are required before these neurodynamic findings can be considered in differential diagnostic research.

## Figures and Tables

**Table 1:** Characterization of the sample included in the study.

	FETTH (*n* = 31)	Migraine (*n* = 33)	Difference
Age, years	19.6 ± 4.3	21.5 ± 1.4	−1.9 (−3.5; −0.03) *p* = 0.032
Females, %	100	69.7	*p* < 0.001
Time since onset, months	33.4 ± 21.4	47.9 ± 23.1	−14.6 (−25.7; −3.4) *p* = 0.011
Episodes per month, n	7.7 ± 3.5	9.9 ± 5.6	−2.2 (−4.5; 0.2) *p* = 0.066
Mean pain intensity per attack (NPRS)	5.4 ± 1.1	5.4 ± 1.4	0.1 (−0.6; 0.7) *p* = 0.846
Headache Disability Index, 0–100	29.2 ± 9.7	40.1 ± 20.8	−10.9 (−19.0; −2.8) *p* = 0.009

Abbreviations: FETTH, frequent episodic tension-type headache; NPRS, Numeric Pain Rating Scale.

**Table 2:** Neurodynamic test response differences between participants with FETTH and migraine.

	FETTH (*n* = 31)	Migraine (*n* = 33)	Adjusted Difference (95% CI)	F	Unadjusted *p* Value	Holm-Adjusted *p* Value	ηp^2^
Long Seated Slump Test							
Sacral flexion, °†	6.6 ± 7.4	−9.3 ± 9.8	13.56 (8.58; 18.54)	29.69	<0.001	<0.001	0.331
Cervical flexion, °†	78.6 ± 16.7	81.8 ± 15.3	−7.58 (−16.48; 1.32)	2.90	0.094	1.000	0.046
Pain at maximal cervical flexion, NPRS	7.2 ± 2.1	8.5 ± 1.7	−1.45 (−2.42; −0.48)	8.94	0.004	0.088	0.128
Slump Test							
Dominant lower limb							
Active knee extension, °	54.6 ± 11.6	52.7 ± 13.1	2.05 (−4.43; 8.53)	0.40	0.529	1.000	0.007
Pain at maximal active knee extension, NPRS	6.3 ± 1.8	7.1 ± 2.2	−1.02 (−2.07; 0.04)	3.73	0.058	1.000	0.058
Non-dominant lower limb							
Active knee extension, °	55.2 ± 13.0	51.8 ± 11.8	3.98 (−2.48; 10.44)	1.52	0.223	1.000	0.024
Pain at maximal active knee extension, NPRS	6.5 ± 2.0	7.3 ± 2.1	−1.01 (−2.07; 0.05)	3.65	0.061	1.000	0.056
Straight Leg Raise							
Dominant lower limb							
Ankle at 30° plantar flexion							
SLR angle at low-pain point (O_1_), °†	48.9 ± 13.0	50.0 ± 10.8	−1.47 (−8.13; 5.19)	0.19	0.661	1.000	0.003
Pain intensity at O_1_, NPRS	1.6 ± 0.8	2.0 ± 1.3	−0.35 (−0.91; 0.21)	1.56	0.217	1.000	0.025
SLR angle at high-pain point (O_2_), °†	71.3 ± 15.0	72.9 ± 15.4	−4.94 (−13.34; 3.47)	1.38	0.245	1.000	0.023
Pain intensity at O_2_, NPRS	8.6 ± 0.6	8.7 ± 0.9	−0.24 (−0.62; 0.14)	1.55	0.217	1.000	0.025
Ankle at 0° plantar flexion							
SLR angle at low-pain point (O_1_), °†	42.3 ± 13.1	45.2 ± 12.1	−4.10 (−11.20; 3.00)	1.34	0.252	1.000	0.022
Pain intensity at O_1_, NPRS	1.7 ± 0.8	2.0 ± 1.3	−0.25 (−0.83; 0.32)	0.76	0.386	1.000	0.012
SLR angle at high-pain point (O_2_), °†	59.6 ± 14.7	65.5 ± 17.2	−10.17 (−18.95; −1.39)	5.37	0.024	0.503	0.082
Pain intensity at O_2_, NPRS	8.7 ± 0.6	8.9 ± 0.9	−0.37 (−0.76; 0.02)	3.58	0.063	1.000	0.055
Non-dominant lower limb							
Ankle at 30° plantar flexion							
SLR angle at low-pain point (O_1_), °†	48.4 ± 13.7	48.8 ± 15.8	−1.46 (−9.50; 6.58)	0.13	0.718	1.000	0.002
Pain intensity at O_1_, NPRS	1.7 ± 0.8	1.8 ± 1.3	−0.04 (−0.59; 0.52)	0.02	0.899	1.000	<0.001
SLR angle at high-pain point (O_2_), °†	69.1 ± 13.8	72.2 ± 18.3	−7.44 (−15.98; 1.09)	3.05	0.086	1.000	0.048
Pain intensity at O_2_, NPRS	8.7 ± 0.7	8.0 ± 2.2	0.69 (−0.19; 1.56)	2.48	0.121	1.000	0.039
Ankle at 0° plantar flexion							
SLR angle at low-pain point (O_1_), °	41.8 ± 12.9	44.3 ± 14.0	−0.68 (−7.53; 6.16)	0.04	0.842	1.000	0.001
Pain intensity at O_1_, NPRS	1.7 ± 0.8	2.0 ± 1.3	−0.29 (−0.85; 0.26)	1.10	0.298	1.000	0.018
SLR angle at high-pain point (O_2_), °†	58.7 ± 14.7	64.2 ± 18.2	−8.95 (−18.18; 0.28)	3.76	0.057	1.000	0.059
Pain intensity at O_2_, NPRS	8.8 ± 0.6	8.9 ± 0.8	−0.20 (−0.57; 0.17)	1.17	0.284	1.000	0.019

Data are presented as mean ± standard deviation. Adjusted differences are expressed as FETTH minus migraine and were adjusted for age, headache duration, and HDI. Unadjusted *p* values correspond to the exploratory ANCOVA analyses. Holm-adjusted *p* values were calculated across all 23 neurodynamic ANCOVA comparisons as a multiplicity-adjusted sensitivity analysis. † Additionally adjusted for sex because significant sex-related differences were observed within the migraine group. Abbreviations: FETTH, frequent episodic tension-type headache; NPRS, Numeric Pain Rating Scale; SLR, Straight Leg Raise; O_1_, initial pain onset (0–3/10 NPRS); O_2_, high/maximal pain (8–10/10 NPRS).

**Table 3:** Exploratory same-sample ROC derivation analysis of neurodynamic findings for discrimination between migraine and FETTH.

	ROC Value	95% CI	Cut-Off Point	*p* Value	Sensitivity (95% CI)	Specificity (95% CI)	Youden Index	Positive LR (95% CI)	Negative LR (95% CI)
Long Seated Slump Test									
Sacral flexion, °†	0.881	0.797–0.965	≤−1.00	<0.001	0.788 (0.611–0.910)	0.903 (0.742–0.980)	0.691	8.12 (3.07–24.00)	0.23 (0.12–0.46)
Cervical flexion, °	0.548	0.406–0.690	≥69.50	0.509	0.848 (0.681–0.949)	0.258 (0.119–0.446)	0.107	1.14 (0.88–1.53)	0.59 (0.22–1.60)
Pain at maximal cervical flexion, NPRS	0.712	0.587–0.838	≥7.85	0.001	0.818 (0.645–0.930)	0.516 (0.331–0.698)	0.334	1.69 (1.17–2.63)	0.35 (0.16–0.78)
Slump Test									
Dominant lower limb									
Active knee extension, °†	0.552	0.410–0.694	≤60.50	0.475	0.788 (0.611–0.910)	0.355 (0.192–0.546)	0.143	1.22 (0.89–1.74)	0.60 (0.27–1.35)
Pain at maximal active knee extension, NPRS	0.652	0.514–0.790	≥6.25	0.031	0.788 (0.611–0.910)	0.548 (0.360–0.727)	0.336	1.74 (1.17–2.78)	0.39 (0.19–0.80)
Non-dominant lower limb									
Active knee extension, °†	0.561	0.418–0.704	≤63.50	0.403	0.939 (0.798–0.993)	0.258 (0.119–0.446)	0.197	1.27 (1.02–1.67)	0.24 (0.05–1.02)
Pain at maximal active knee extension, NPRS	0.628	0.490–0.766	≥7.25	0.069	0.576 (0.392–0.745)	0.677 (0.486–0.833)	0.253	1.78 (1.02–3.30)	0.63 (0.39–1.00)
Straight Leg Raise									
Dominant lower limb									
Ankle at 30° plantar flexion									
SLR angle at low-pain point (O_1_), °	0.559	0.416–0.702	≥51.50	0.417	0.485 (0.308–0.665)	0.677 (0.486–0.833)	0.162	1.50 (0.82–2.85)	0.76 (0.50–1.15)
Pain intensity at O_1_, NPRS	0.533	0.390–0.677	≥3.35	0.650	0.152 (0.051–0.319)	1.000 (0.888–1.000)	0.152	∞ (1.29–∞)	0.85 (0.73–0.98)
SLR angle at high-pain point (O_2_), °	0.572	0.428–0.717	≥70.50	0.327	0.636 (0.451–0.796)	0.613 (0.422–0.782)	0.249	1.64 (1.00–2.83)	0.59 (0.35–1.01)
Pain intensity at O_2_, NPRS	0.525	0.381–0.669	≥9.85	0.735	0.212 (0.090–0.389)	0.968 (0.833–0.999)	0.180	6.62 (1.14–40.41)	0.81 (0.67–0.98)
Ankle at 0° plantar flexion									
SLR angle at low-pain point (O_1_), °	0.584	0.442–0.725	≥46.00	0.247	0.485 (0.308–0.665)	0.742 (0.554–0.881)	0.227	1.88 (0.97–3.83)	0.69 (0.47–1.03)
Pain intensity at O_1_, NPRS	0.518	0.375–0.661	≥3.15	0.805	0.182 (0.070–0.355)	1.000 (0.888–1.000)	0.182	∞ (1.56–∞)	0.82 (0.70–0.96)
SLR angle at high-pain point (O_2_), °	0.623	0.481–0.765	≥65.50	0.090	0.545 (0.364–0.719)	0.839 (0.663–0.945)	0.384	3.39 (1.52–8.11)	0.54 (0.36–0.81)
Pain intensity at O_2_, NPRS	0.572	0.429–0.716	≥9.85	0.324	0.303 (0.156–0.487)	0.935 (0.786–0.992)	0.239	4.66 (1.28–18.42)	0.75 (0.58–0.95)
Non-dominant lower limb									
Ankle at 30° plantar flexion									
SLR angle at low-pain point (O_1_), °	0.514	0.371–0.657	≥47.50	0.846	0.576 (0.392–0.745)	0.548 (0.360–0.727)	0.124	1.27 (0.79–2.12)	0.77 (0.46–1.29)
Pain intensity at O_1_, NPRS†	0.532	0.388–0.675	≤1.15	0.664	0.576 (0.392–0.745)	0.548 (0.360–0.727)	0.124	1.27 (0.79–2.12)	0.77 (0.46–1.29)
SLR angle at high-pain point (O_2_), °	0.568	0.423–0.713	≥78.00	0.357	0.515 (0.335–0.692)	0.742 (0.554–0.881)	0.257	2.00 (1.04–4.04)	0.65 (0.43–0.98)
Pain intensity at O_2_, NPRS†	0.579	0.438–0.719	≤7.50	0.272	0.182 (0.070–0.355)	1.000 (0.888–1.000)	0.182	∞ (1.56–∞)	0.82 (0.70–0.96)
Ankle at 0° plantar flexion									
SLR angle at low-pain point (O_1_), °	0.566	0.425–0.708	≥45.50	0.357	0.424 (0.255–0.608)	0.742 (0.554–0.881)	0.166	1.64 (0.82–3.42)	0.78 (0.54–1.11)
Pain intensity at O_1_, NPRS	0.547	0.405–0.689	≥2.85	0.517	0.273 (0.133–0.455)	0.839 (0.663–0.945)	0.111	1.70 (0.66–4.45)	0.87 (0.67–1.12)
SLR angle at high-pain point (O_2_), °	0.626	0.485–0.767	≥69.50	0.079	0.455 (0.281–0.636)	0.871 (0.702–0.964)	0.326	3.53 (1.41–9.46)	0.63 (0.45–0.88)
Pain intensity at O_2_, NPRS	0.536	0.393–0.679	≥9.65	0.625	0.242 (0.111–0.423)	0.871 (0.702–0.964)	0.113	1.88 (0.67–5.50)	0.87 (0.69–1.10)

ROC analyses represent exploratory, same-sample derivation analyses. Thresholds correspond to the values maximizing Youden’s index in the present sample and should not be interpreted as validated diagnostic cut-offs. Sensitivity and specificity are reported with 95% exact binomial confidence intervals. Likelihood ratios are reported with 95% confidence intervals derived from the corresponding 2 × 2 classification tables. When no false-positive observations occurred, LR+ was infinite, and its confidence interval is reported with an infinite upper bound. Migraine was considered the positive state. † For variables in which lower values were associated with migraine, the direction of the ROC curve and the corresponding classification rule were reversed.

## Data Availability

The original contributions presented in this study are included in the article. Further inquiries can be directed to the corresponding authors.
